# Reduced Risk of Progression from Non-Severe to Severe COVID-19 in Hospitalized Dialysis Patients by Full COVID-19 Vaccination

**DOI:** 10.3390/jcm11216348

**Published:** 2022-10-27

**Authors:** Mitsuru Ichii, Masafumi Kurajoh, Yujiro Okute, Yasutaka Ihara, Takumi Imai, Tomoaki Morioka, Katsuhito Mori, Tetsuo Shoji, Yoshihiro Tsujimoto, Takanobu Ubai, Masanori Emoto

**Affiliations:** 1 Division of Internal Medicine, Dialysis Center, Inoue Hospital, Suita 564-0053, Japan; 2 Department of Metabolism, Endocrinology and Molecular Medicine, Osaka Metropolitan University Graduate School of Medicine, Osaka 545-8585, Japan; 3 Department of Medical Statistics, Osaka Metropolitan University Graduate School of Medicine, Osaka 545-8585, Japan; 4 Department of Nephrology, Osaka Metropolitan University Graduate School of Medicine, Osaka 545-8585, Japan; 5 Department of Vascular Medicine, Osaka Metropolitan University Graduate School of Medicine, Osaka 545-8585, Japan; 6 Department of Urology, Inoue Hospital, Suita 564-0053, Japan

**Keywords:** COVID-19, full vaccination, severe progression, dialysis patients

## Abstract

Coronavirus disease 2019 (COVID-19) vaccination reduces the risk of progression to severe COVID-19 in the general population. To examine that preventive effect in dialysis patients, the association of vaccination status with severe COVID-19 progression was investigated in this retrospective observational study conducted from December 2020 to May 2022 of 100 such patients hospitalized for non-severe COVID-19 at Inoue Hospital (Suita, Japan). Fifty-seven were fully vaccinated, defined as receiving a COVID-19 vaccine second dose at least 14 days prior to the onset of COVID-19, while 43 were not. Among all patients, 13 (13.0%) progressed to severe COVID-19 with a median (interquartile range) time of 6 (2.5–9.5) days, while 87 (87.0%) were discharged after 11 (8–16) days. Kaplan–Meier analysis showed that fully vaccinated patients had a significantly lower rate of progression to severe COVID-19 (*p* = 0.001, log-rank test). Cox proportional hazard analysis also indicated that full COVID-19 vaccination was significantly associated with reduced instances of progression to severe COVID-19 (hazard ratio 0.104, 95% confidence interval 0.022 to 0.483; *p* = 0.004) after balancing patient background characteristics using an inverse probability of treatment weight method. These results suggest that full vaccination status contributes to reducing the risk of progression from non-severe to severe COVID-19 in dialysis patients.

## 1. Introduction

The number of patients diagnosed with coronavirus disease 2019 (COVID-19), which is caused by severe acute respiratory syndrome coronavirus 2 (SARS-CoV-2), has been increasing throughout the world [1,2,3]. Accordingly, the number of severe COVID-19 cases has also been rising, especially among patients undergoing dialysis [4]. However, along with a high risk of mortality associated with COVID-19, those patients require advanced medical care [4,5], thus the introduction of measures to prevent their progression to severe COVID-19 is needed. 

Several different COVID-19 vaccines have been developed since the establishment of the worldwide pandemic, and vaccination treatments have been reported to be effective to reduce the severity as well as COVID-19-related mortality in general populations [6,7,8]. In Japan, the Pfizer-BioNTech COVID-19 vaccine was approved in February 2021, followed by the Moderna and Oxford-AstraZeneca vaccines in May 2021. Vaccination began sequentially in February 2021 for healthcare workers, then in June 2021 for individuals with underlying medical conditions, including dialysis patients, with public and private sectors working together to promote vaccination. Although the immune response to such vaccine treatment has been reported to be compromised in dialysis patients [9,10,11], vaccination has also been shown to reduce the rate of non-severe COVID-19 cases as well as COVID-19-related mortality in patients receiving that treatment [12,13,14,15,16,17]. However, few studies have assessed a dialysis patient cohort regarding the relationship of vaccination with the progression to severe COVID-19. 

To determine whether COVID-19 vaccination reduces the risk of progression from non-severe to severe COVID-19 in dialysis patients, the present study examined the association of fully vaccinated status with progression to severe COVID-19 in such patients hospitalized for non-severe COVID-19 at Inoue Hospital, which has been designated by the Osaka Prefecture government as a priority medical institution for receiving dialysis patients with non-severe status who are unable to receive treatment at their local dialysis hospital or clinic.

## 2. Materials and Methods

### 2.1. Study Design and Participants

This retrospective observational study was performed at Inoue Hospital (Suita, Japan) to examine the association of fully vaccinated status with progression from non-severe (mild, moderate I, moderate II) to severe COVID-19 based on specific patient criteria, as follows. Inclusion criteria: (1) admitted to Inoue hospital for non-severe COVID-19 from 21 December 2020 to 18 May 2022, (2) undergoing dialysis, and (3) age ≥ 20 years. Exclusion criteria: (1) with severe COVID-19 on admission, (2) not undergoing dialysis, and (3) transferred to our hospital from a tertiary hospital after improvement from severe to non-severe COVID-19 status. 

The target of the primary analysis was all patients who met the criteria. A sensitivity analysis for patients hospitalized before December 2021, prior to the emergence of the Omicron strain, which has been reported to have less severe disease progression as compared to other virus strains [18], was also performed.

### 2.2. Full COVID-19 Vaccination

Vaccination history including date and number was confirmed by reviewing vaccination certificates, the receipt of a referral letter to our hospital, and/or interview findings. Full COVID-19 vaccination was defined as receiving a second dose of a COVID-19 vaccine at least 14 days prior to the onset of COVID-19. Not full COVID-19 vaccination was defined as no history of such vaccination (unvaccinated) or the receipt of only one dose or if the second dose was received within 14 days of the onset of COVID-19 (partially vaccinated) [19,20].

### 2.3. Diagnosis of COVID-19

The diagnosis of COVID-19 for each patient was performed using clinical practice guidelines for COVID-19 published by the Japanese Ministry of Health, Labour and Welfare [21,22,23]. In brief, COVID-19 was confirmed by a nucleic acid amplification test, which included real-time polymerase chain reaction (PCR), transcription-mediated amplification, and loop-mediated isothermal amplification assay findings, as well as results of a qualitative antigen test or a quantitative test for SARS-CoV-2. 

### 2.4. COVID-19 Severity Classification

The severity of COVID-19 was classified based on oxygenation and respiratory symptoms as mild when percutaneous oxygen (SpO_2_) saturation was ≥96% and there were no respiratory symptoms or only coughing without shortness of breath; moderate I in patients not suffering from respiratory failure who showed SpO_2_ saturation from 93% to 96%, shortness of breath, or pneumonia findings; or moderate II in patients suffering from respiratory failure who showed SpO_2_ ≤ 93% or required O_2_ administration. A severe classification was noted upon admission to the intensive care unit (ICU) or when the use of a mechanical ventilator was required [21,22,23]. Thus, patients classified as mild, moderate I, or moderate II were defined as non-severe COVID-19 [24]. 

### 2.5. Management of COVID-19 during Hospitalization

Guidelines current at the time were utilized to treat patients while hospitalized [21,22,23]. For discharge after the confirmation of COVID-19 diagnosis, the following criteria were referred to: (i) onset of symptoms at least 10 days prior and symptom resolution at least 72 h prior, or (ii) 24 h after symptom resolution with negative results shown in PCR or quantitative antigen tests performed twice with 24 h or more between tests [21,22,23]. Patients who progressed from non-severe to severe COVID-19 were transferred to a tertiary hospital that had an ICU available, unless life-prolonging treatment was not required.

### 2.6. Outcome

The examined outcome of the present study was the time (days) between hospital admission and progression to severe COVID-19. Patients who did not show a progression to severe status and met the criteria for discharge were assumed to not have progressed to severe status after discharge, as previously described [24].

### 2.7. Other Clinical Parameters

Information regarding present and past illness, including cardiovascular disease and chronic respiratory disease, body weight, height, use of medication, and date of onset of COVID-19, based on symptom onset, for each enrolled patient was obtained from medical records. Diabetes, hypertension, and dyslipidemia were diagnosed based on the previous history of treatment for the respective condition or guidelines provided by the Japanese Diabetes Society, Japanese Society of Hypertension, or Japan Atherosclerosis Society [25,26,27]. Abnormal findings indicating pneumonia were evaluated using a chest computed tomography examination [24].

### 2.8. Statistical Analysis 

Baseline demographics and clinical characteristics are presented as medians (interquartile range (IQR)) for continuous variables and numbers (percentages) for categorical variables, with differences in characteristics based on vaccination status evaluated by absolute standardized difference (ASD), as well as Mann–Whitney’s U test (continuous variables) and a chi-squared test (categorical variables). The numbers and percentages of patients with severe COVID-19 progression are presented, with the number needing treatment (NNT) estimated based on risk difference (RD) for severe COVID-19 progression between groups (NNT = 1/RD). As a primary analysis, to investigate the association of full COVID-19 vaccination with severe COVID-19 progression, the probability of no progression to severe COVID-19 after hospital admission was estimated using the Kaplan–Meier method in groups determined by vaccination status and compared using log-rank test results. To address the problem of confounding because of differences in the background characteristics of the groups, Cox proportional hazard analysis was performed with a propensity score method. Propensity scores were estimated by logistic regression using patient background characteristics, while the hazard ratio (HR) of patients with full vaccination status to progress to severe COVID-19 was estimated using an inverse probability of treatment weight (IPTW) method. The following characteristics were considered as potential confounding factors: age; body mass index (BMI); gender; duration of dialysis; the presence of diabetes, hypertension, dyslipidemia, cardiovascular disease, and/or chronic respiratory disease; the use of an angiotensin-converting enzyme (ACE) inhibitor/angiotensin II receptor blocker (ARB); days from onset of disease to hospitalization; and severity of COVID-19 at time of admission. The balance of patient background characteristics in IPTW analysis was assessed by ASD. For sensitivity analysis, the association of vaccination status with severe COVID-19 progression was examined in patients hospitalized before December 2021, prior to the emergence of the Omicron strain. Due to a limited number of fully vaccinated cases, a simple Cox proportional hazard analysis as well as the Kaplan–Meier method were used. Results obtained by including partial vaccination cases in the vaccination group were also explored.

All statistical analyses were performed using the R software package, version 4.0.5 (R Foundation for Statistical Computing, Vienna, Austria). An ASD < 0.1 was considered to indicate a balance between groups [28]. A two-sided *p*-value of <0.05 was used to indicate statistical significance. 

## 3. Results

### 3.1. Study Population

During the study period, 106 patients with non-severe COVID-19 were admitted to Inoue Hospital. Any patient transferred from a tertiary hospital after improvement from severe to non-severe status (*n* = 3), as well as non-dialysis patients with COVID-19 (*n* = 3), were excluded from the present analysis. As a result, 100 dialysis patients, of whom 96 were undergoing hemodialysis and 4 peritoneal dialysis, with non-severe COVID-19, were analyzed. Of these, 57 patients were fully vaccinated, while 43 were not (36 unvaccinated, 7 partial vaccination). Furthermore, prior to December 2021 (before the emergence of the Omicron strain), 47 dialysis patients were identified, of whom 10 were fully vaccinated and 37 were not (32 unvaccinated, 5 partial vaccination).

### 3.2. Clinical Characteristics of Dialysis Patients, and Comparisons between Those with and without Fully Vaccinated Status

The characteristics of all enrolled patients (*n* = 100) are presented in Table 1. The number of patients with severity noted as mild, moderate I, and moderate II COVID-19 was 41 (41.0%), 48 (48.0%), and 11 (11.0%), respectively. BMI, the presence of diabetes, hypertension, or cardiovascular disease, the use of ACE inhibitors/ARBs, and COVID-19 severity at the time of admission were not well balanced between the fully and not fully vaccinated groups (Table 1).

### 3.3. Medications during Hospitalization Used for COVID-19 

Medications administered for COVID-19 included antivirals in 58 (58.0%) patients, neutralizing antibodies in 53 (53.0%), and steroids in 16 (16.0%). In the fully vaccinated group, medications given for COVID-19 included antivirals in 33 (57.9%), neutralizing antibodies in 46 (80.7%), and steroids in 1 (1.8%), while in the without full vaccination group, these were antivirals in 25 (58.1%), neutralizing antibodies in 7 (16.3%), and steroids in 15 (34.9%) patients. As for patients who progressed to severe COVID-19, medications administered for COVID-19 included antivirals in 12 (92.3%), neutralizing antibodies in 1 (7.7%), and steroids in 11 (84.6%), while in those who did not progress to severe COVID-19, medications administered for COVID-19 included antivirals in 46 (52.9%), neutralizing antibodies in 52 (59.8%), and steroids in 5 (5.7%).

### 3.4. Progression from Non-Severe to Severe COVID-19

**Figure 1:**
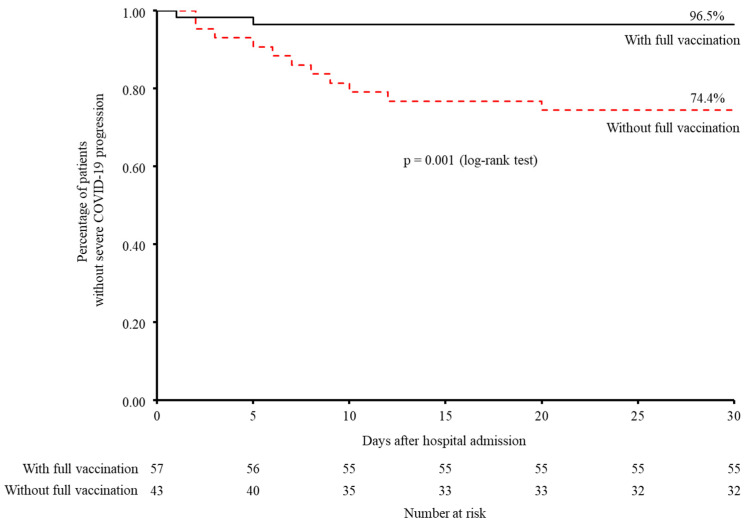
Percentage of patients without progression to severe COVID-19 stratified by vaccination status estimated by the Kaplan–Meier method. Abbreviations: COVID-19, coronavirus disease 2019.

**Figure 2:**
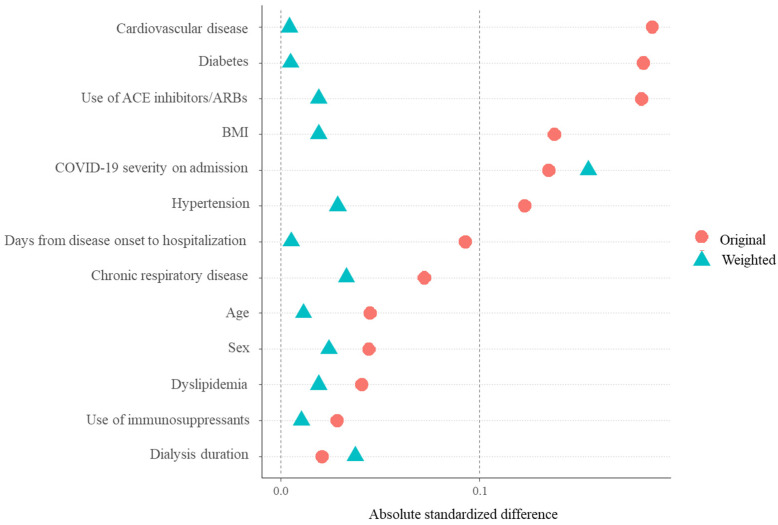
Absolute standardized differences in patients with and without full vaccination before and after IPTW. Abbreviations: IPTW, inverse probability of treatment weight; ACE, angiotensin-converting enzyme; ARB, angiotensin II receptor blocker; BMI, body mass index; COVID-19, coronavirus disease 2019.

Of the present 100 dialysis patients with non-severe COVID-19 at the time of hospitalization, 13 (13.0%) progressed to severe status and 87 (87.0%) were discharged. Among the 13 who showed a progression to severe status, the median time from hospitalization to severe progression was 6 days (IQR, 2.5–9.5; range, 1–20), and this included two (3.5%) in the fully vaccinated group and eleven (25.6%) in the not fully vaccinated group. For patients discharged from the hospital, the median time from hospitalization to discharge was 11 days (IQR, 8–16; range, 5–41). The NNT to prevent progression to severe COVID-19 with full COVID-19 vaccination was 4.53. Kaplan–Meier analysis showed that fully vaccinated patients had a significantly lower rate of severe progression (*p* = 0.001 by log-rank test) (Figure 1). Additionally, Cox proportional hazard analysis indicated that full COVID-19 vaccination status was significantly associated with reduced progression to severe COVID-19 (HR 0.104, 95% confidence interval (CI) 0.022 to 0.483; *p* = 0.004), after balancing the patient background characteristics with an IPTW method, except for COVID-19 severity on admission (Figure 2). When COVID-19 severity at the time of admission was added to the above-mentioned Cox proportional hazard model, full COVID-19 vaccination retained a significant association with a reduced rate of progression to severe COVID-19 (HR 0.096, 95% CI 0.020 to 0.465; *p* = 0.004). The results of the sensitivity analysis of patients admitted before December 2021 showed that full COVID-19 vaccination and full/partial vaccination status had trends similar to the primary results (HR 0.322, 95% CI 0.041 to 2.493: *p* = 0.278 and HR 0.175, 95% CI 0.023 to 1.354: *p* = 0.095, respectively) (Figure S1).

## 4. Discussion

In the present study, full COVID-19 vaccination status was shown to be significantly associated with a reduced incidence of the progression to a severe condition in dialysis patients hospitalized for non-severe COVID-19. These results strongly suggest that full COVID-19 vaccination contributes to reducing the risk of progression from non-severe to severe COVID-19 in patients undergoing dialysis.

Full COVID-19 vaccination has been reported to reduce the severity and mortality of COVID-19 in general populations [6,7,8]. As for dialysis patients, full vaccination status has been shown to reduce conditions related to non-severe COVID-19, such as the need for hospitalization and oxygen administration, as well as COVID-19-related mortality [12,13,14,15,16,17]. However, studies regarding the association of such vaccination with severe COVID-19 progression in dialysis patients are limited. Toda et al. reported that full vaccination status was associated with reduced disease severity, including severe COVID-19, in dialysis patients, though the number of cases investigated was small (*n* = 26) and the association was not adjusted using known risk factors [11]. In another study, Ashby et al. noted that full vaccination status reduced the need for intubation, shown to correspond to severe COVID-19, in dialysis patients after adjustment for known risk factors including age and diabetes, though the difference was not significant [12]. Furthermore, that association was not adjusted by COVID-19 severity on admission, which is known to be deeply related to severe COVID-19 progression. The results of the present study demonstrated a significant association of full vaccination status with reduced progression from non-severe to severe COVID-19, independent of patient background characteristics including the severity of the disease at the time of admission. Together, the present results, along with those previous studies, suggest that the completion of a full COVID-19 vaccination protocol is a significant factor in inhibiting advancement to any stage during a clinical course associated with COVID-19 progression in dialysis patients as well as the general population.

Dialysis patients have been reported to have a poor immune response to vaccines against viruses such as influenza and hepatitis B [29,30,31] and also a poor immune response to vaccines against COVID-19 [9,10,11]. However, in the present study, the NNT for full vaccination to prevent severe COVID-19 progression was approximately 4.5, and the HR of full vaccination for severe COVID-19 progression was approximately 0.1, indicating a marked prevention effect of full COVID-19 vaccination on the progression to severe COVID-19, even in dialysis patients. Unfortunately, the efficacy of full COVID-19 vaccination status in the suppression of severe COVID-19 progression in dialysis as compared with non-dialysis patients was not examined in the present study, and there was also no evaluation of COVID-19 vaccine-induced immune response. Therefore, the relationship between the degree of immune response to a COVID-19 vaccine with the reduction in severe COVID-19 progression in dialysis patients requires further investigation.

The present study has some important limitations. First, the number of patients enrolled was low. Inoue Hospital has been accepting dialysis patients with non-severe COVID-19 from throughout Osaka Prefecture when they are unable to receive treatment at their local dialysis hospital or dialysis clinic. Thus, the number of patients admitted is dependent on the rate of incidence of COVID-19 and/or acceptance at the dialysis hospital or dialysis clinic where the patient is receiving treatment, resulting in the low number in the present study. Second, while a survey of SARS-CoV-2 strains was not conducted, to account for the effect of the predominant strain, we examined the association of vaccination status with severe COVID-19 progression in patients hospitalized before December 2021, prior to the emergence of the Omicron strain, which has been reported to have less severe disease progression as compared to other strains [32]. Full COVID-19 vaccination status and full/partial vaccination status showed similar trends as compared with the primary results (Figure S1). However, due to the low number of cases, a full analysis including patient background could not be performed. In addition, since many patients were fully vaccinated after December 2021, it was not possible to conduct a sensitivity analysis of the association of full vaccination with severe COVID-19 in patients admitted after December 2021. Furthermore, the number of patients who developed severe status was extremely limited in the analyses that focused on the two-dose only and three-dose vaccination (booster) groups (2/43 and 0/14, respectively), making it difficult to investigate the association of severe progression in those groups. Thus, large prospective studies are needed to investigate the association of vaccination status, including single-dose and booster-dose administration, with severe COVID-19 progression seen with different coronavirus strains. Third, the specific type of COVID-19 vaccine administered was not fully investigated. Fourth, dialysis adequacy, anuria status, and infectious diseases, such as those caused by the human immunodeficiency virus, which is known to strongly influence the clinical course of COVID-19-positive patients, were not investigated. Finally, the COVID-19 severity classification defined by clinical practice guidelines presented and utilized in Japan [21,22,23] is not fully consistent with the classification used in other countries, though severe COVID-19 status noted in the Japanese criteria basically corresponds to critical COVID-19 elsewhere [33,34]. 

## 5. Conclusions

The present results showed that full COVID-19 vaccination was significantly associated with reduced progression to severe COVID-19 in dialysis patients with non-severe COVID-19. It is thus suggested that full vaccination status reduces the risk of the progression of dialysis patients from a non-severe to a severe disease condition.

## Figures and Tables

**Table 1:** Clinical characteristics of dialysis patients with non-severe COVID-19 (*n* = 100), and comparisons between those with and without full vaccination.

	Total (*n* = 100)	With Full Vaccination (*n* = 57)	Without Full Vaccination (*n* = 43)	ASD	*p* Value
Age, years	71.0 (62.0–76.0)	72.0 (62.0–78.0)	70.0 (63.0–74.0)	0.045	0.482
Male	64 (64)	37 (64.9)	27 (62.8)	0.044	0.827
BMI, kg/m^2^	22.0 (19.5–24.4)	21.1 (19.0–24.2)	22.6 (20.6–24.4)	0.136	0.193
Dialysis duration, years	6.0 (2.8–15.3)	6.0 (2.0–16.0)	7.0 (3.0–15.0)	0.020	0.727
Diabetes	46 (46)	24 (42.1)	22 (51.2)	0.182	0.368
Hypertension	81 (81)	45 (78.9)	36 (83.7)	0.123	0.547
Dyslipidemia	50 (50)	28 (49.1)	22 (51.2)	0.041	0.84
Cardiovascular disease	70 (70)	42 (73.7)	28 (65.1)	0.187	0.355
Chronic respiratory disease	13 (13)	8 (14.0)	5 (11.6)	0.072	0.723
Use of ACE inhibitors/ARBs	47 (47)	29 (50.9)	18 (41.9)	0.182	0.371
Use of immunosuppressants	5 (5)	3 (5.3)	2 (4.7)	0.028	0.889
Days from disease onset to hospitalization	3.0 (1.0–4.0)	3.0 (1.0–3.0)	3.0 (1.0–5.0)	0.092	0.774
COVID-19 severity on admission				0.135	0.802
Mild	41 (41)	22 (38.6)	19 (44.2)		
Moderate I	48 (48)	29 (50.9)	19 (44.2)		
Moderate II	11 (11)	6 (10.5)	5 (11.6)		

Values are expressed as numbers (%) or medians (interquartile range) unless otherwise noted. Differences in characteristics stratified by those with and without fully vaccinated status were evaluated by ASD, as well as Mann–Whitney’s U test (continuous variables) and a chi-squared test (categorical variables). Abbreviations: ACE, angiotensin-converting enzyme; ARB, angiotensin II receptor blocker; ASD, absolute standardized difference; BMI, body mass index; COVID-19, coronavirus disease 2019.

## Data Availability

The data presented in this study are available on request from the corresponding author. The data are not publicly available due to ethics committee permission.

## Supplementary Materials

The supporting information can be downloaded at: https://www.mdpi.com/article/10.3390/jcm11216348/s1, Figure S1: Percentage of patients admitted before December 2021 without progression to severe COVID-19 stratified based on (A) with or without full vaccination and (B) full/partial vaccination status. Estimations were performed using the Kaplan–Meier method.
