# Supplementary material for: Reduced Risk of Progression from Non-Severe to Severe COVID-19 in Hospitalized Dialysis Patients by Full COVID-19 Vaccination
Source: J Clin Med. 2022 Oct 27;11(21):6348. doi: 10.3390/jcm11216348 (PMC9657170; doi:10.3390/jcm11216348)
Supplement: Supplementary file 1 [file jcm-11-06348-s001.zip › jcm-1921430-supplementary.pptx]

## Slide 1
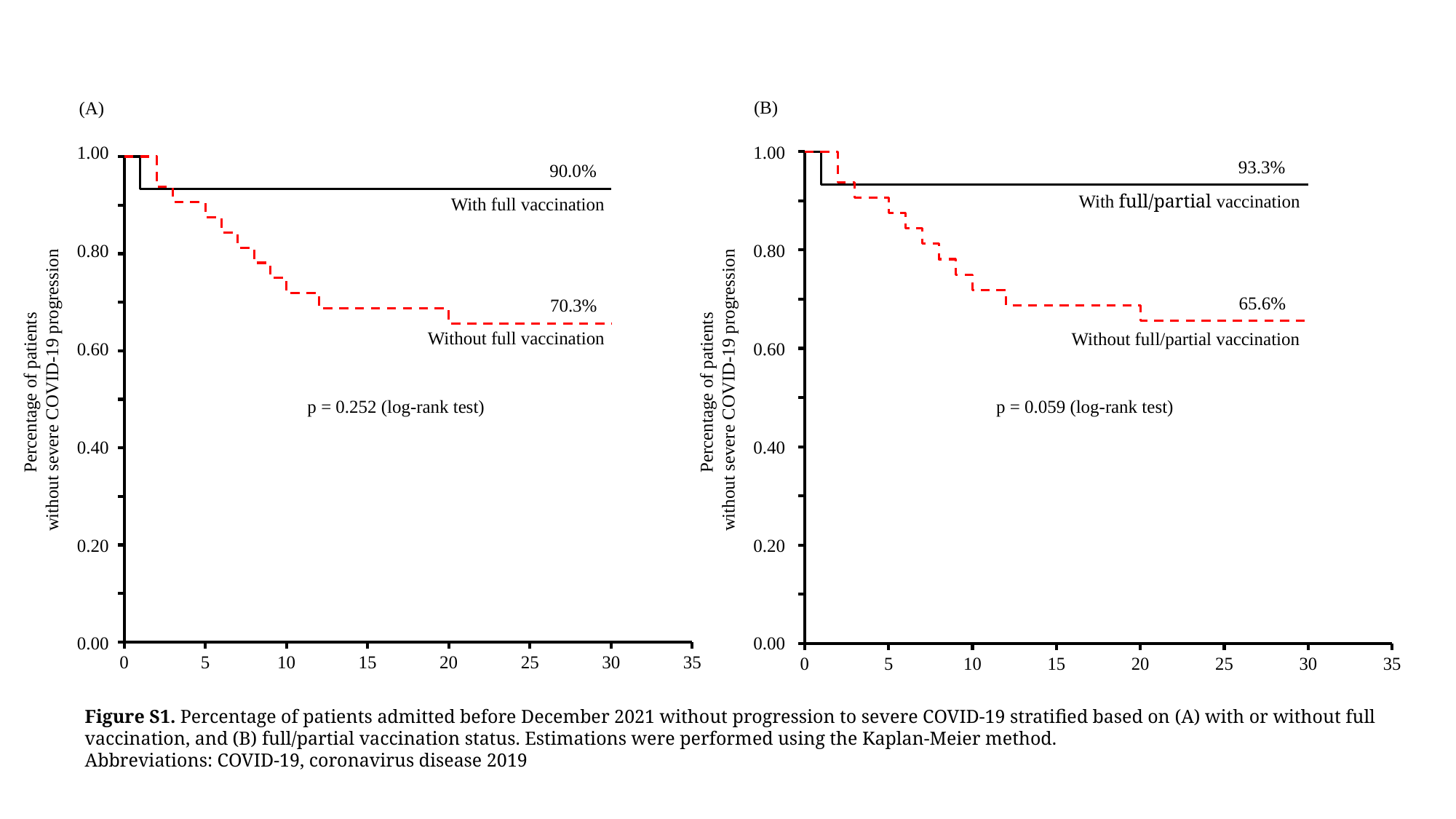

(B)
(A)
### Chart
| Category | | |
|---|---|---|
### Chart
| Category | | |
|---|---|---|1.00
1.00
93.3%
90.0%
With full/partial vaccination
With full vaccination
0.80
0.80
65.6%
70.3%
Without full vaccination
Without full/partial vaccination
0.60
0.60
Percentage of patients
 without severe COVID-19 progression
Percentage of patients
 without severe COVID-19 progression
p = 0.252 (log-rank test)
p = 0.059 (log-rank test)
0.40
0.40
0.20
0.20
0.00
0.00
Figure S1. Percentage of patients admitted before December 2021 without progression to severe COVID-19 stratified based on (A) with or without full vaccination, and (B) full/partial vaccination status. Estimations were performed using the Kaplan-Meier method.
Abbreviations: COVID-19, coronavirus disease 2019
